# P-420. Trends in Treatment of Methicillin-Resistant Staphylococcus aureus (MRSA) in Hospitalized Children

**DOI:** 10.1093/ofid/ofaf695.636

**Published:** 2026-01-11

**Authors:** Sirey Zhang, Adam Hersh, Sonia Mehra, Jared Olson

**Affiliations:** University of Utah, Salt Lake City, UT; University of Utah, Salt Lake City, UT; University of Utah, Salt Lake City, UT; Intermountain Health, Salt Lake City, Utah

## Abstract

**Background:**

Methicillin-Resistant *Staphylococcus aureus* (MRSA) causes serious infections and is linked to poor outcomes. Although vancomycin is a first-line therapy, salvage therapy with alternatives to vancomycin or combination therapy is often considered for patients with persistent bacteremia. Newer antibiotics have expanded treatment options, but data on their use and variability within pediatrics remain limited. We performed this retrospective study to evaluate changes in MRSA antibiotic treatment choices over time in children.

**Figure 1:**
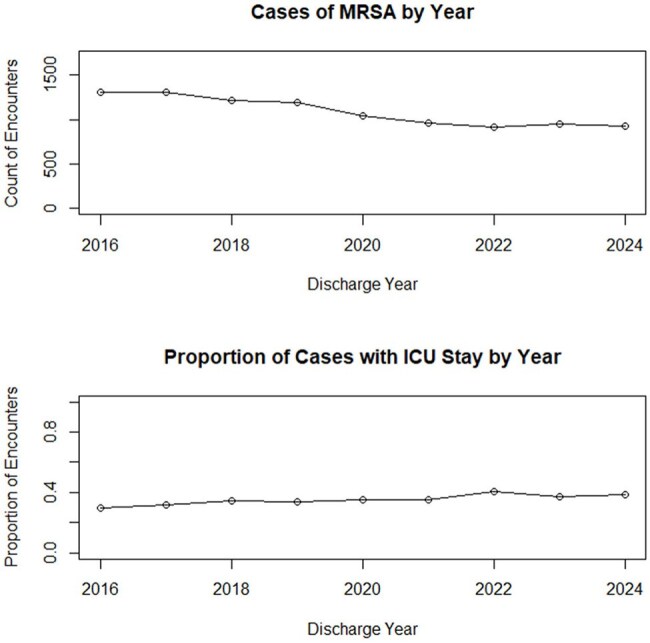
Encounters and severity by year.

**Figure 2:**
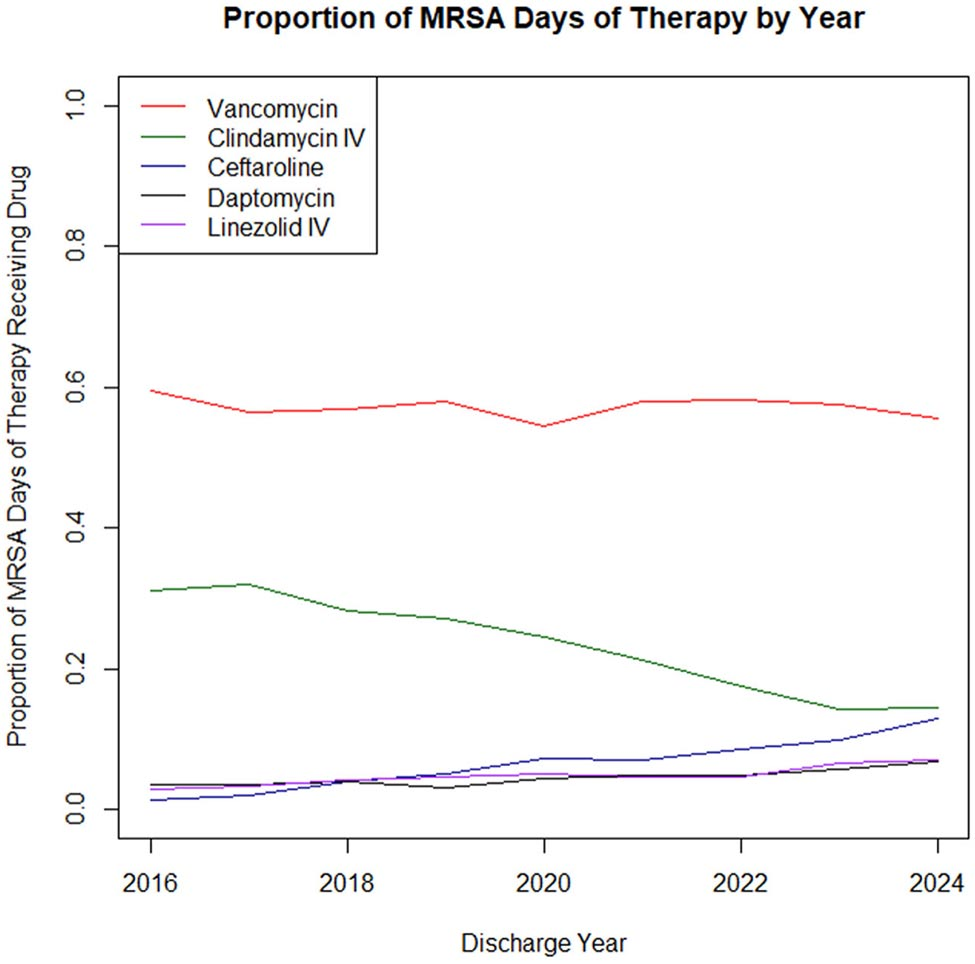
MRSA therapy trends by year.

**Methods:**

We used ICD-10 codes to identify MRSA infections in the Pediatric Health Information System database between January 2016 to December 2024 from children’s hospitals who contributed data for the entire period. Patients were included if hospitalized for a minimum of 3 days and started on anti-MRSA antibiotics (vancomycin, IV clindamycin, ceftaroline, daptomycin, and IV linezolid) within the first 3 hospital days. We excluded patients with Cystic Fibrosis, in the newborn intensive care unit, and receiving only Trimethoprim/sulfamethoxazole. We calculated the number of hospital discharges involving MRSA, including ICU admissions. We also determined the proportion of anti-MRSA days for each antibiotic and used a Chi-square test to compare antibiotic use between 2016 and 2024.

**Figure 3:**
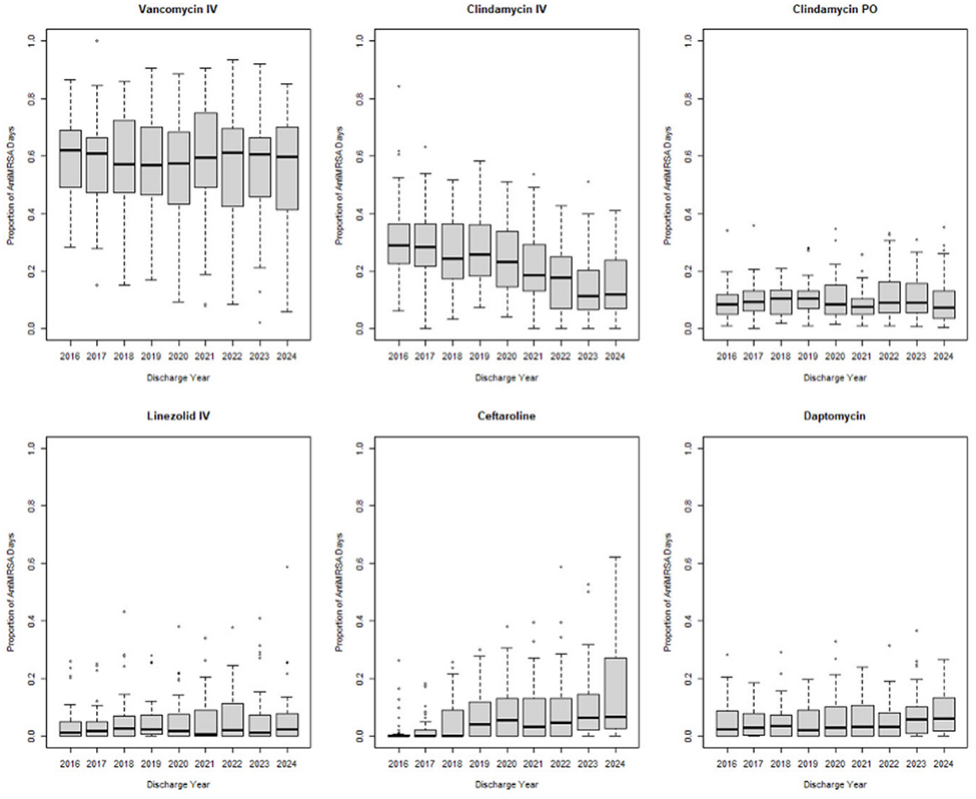
Boxplot showing variability of anti MRSA agents over 40 hospitals.

**Figure 4.**
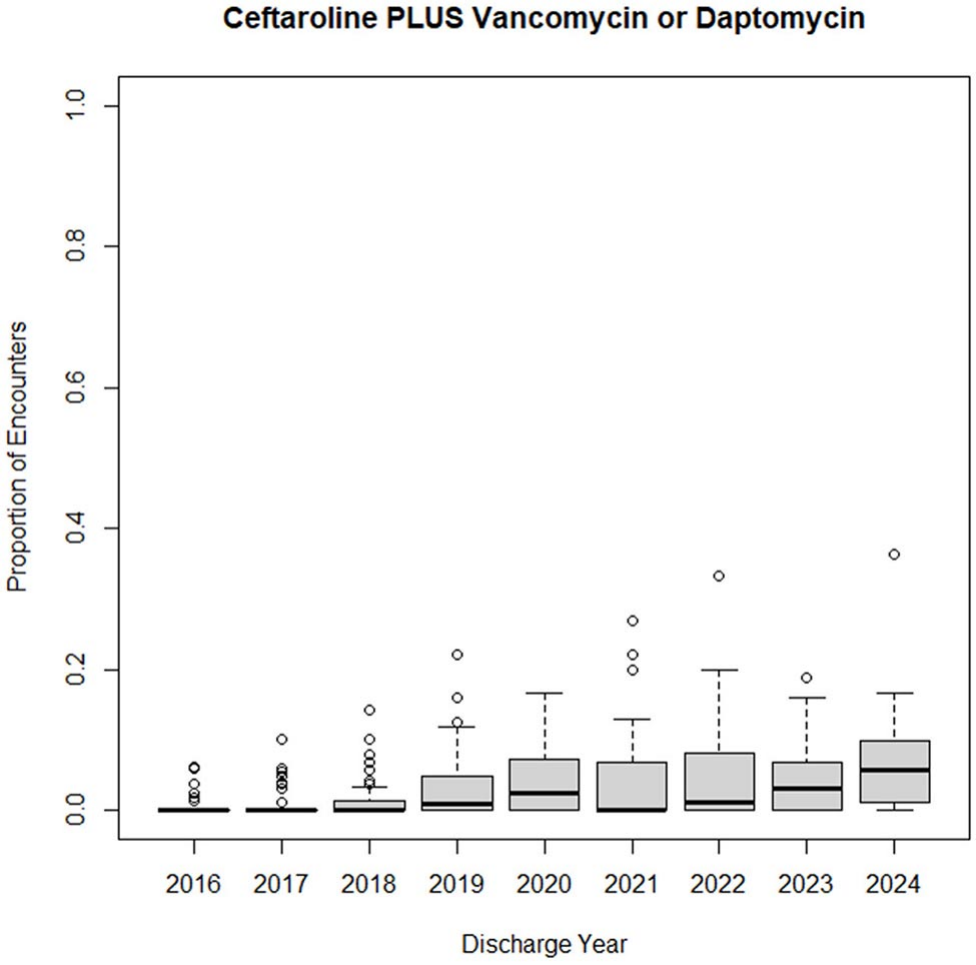
Boxplot showing distribution of ceftaroline combination therapy use among hospitals by year.

**Results:**

We analyzed 16,875 encounters from 15,251 patients across 40 children's hospitals, totaling 169,414 MRSA therapy days from 2016–2024 (Figure 1). MRSA hospitalizations fell from 1303 to 932 annually, while ICU admissions rose from 29% to 38% (p< 0.001). Vancomycin (59% to 56%) and IV clindamycin (31% to 14%) use declined, whereas ceftaroline (1% to 13%), IV linezolid (3% to 7%), and daptomycin (4% to 7%) use increased (all p< 0.001) (Figure 2). Combination therapy with ceftaroline and daptomycin or vancomycin rose from 0% to 4% (p< 0.001). Substantial variation in antibiotic use was observed across hospitals (Figures 3, 4).

**Conclusion:**

Since 2016, MRSA hospitalizations have declined, although ICU admissions have risen. Use of vancomycin and IV clindamycin is decreasing as newer agents become more common. Given variation across hospitals, further research is needed to identify the most effective treatment strategies.

**Disclosures:**

All Authors: No reported disclosures

